# Epigenetic alteration by DNA-demethylating treatment restores apoptotic response to glucocorticoids in dexamethasone-resistant human malignant lymphoid cells

**DOI:** 10.1186/1475-2867-14-35

**Published:** 2014-04-23

**Authors:** Aaron L Miller, Chuandong Geng, Georgiy Golovko, Meenakshi Sharma, Jason R Schwartz, Jiabin Yan, Lawrence Sowers, William R Widger, Yuriy Fofanov, Wayne V Vedeckis, E Brad Thompson

**Affiliations:** 1 Department of Biochemistry & Molecular Biology, (ALM present address, Department. of Pediatrics, & Assay Devel. Service Division Galveston National Lab.), University of Texas Medical Branch, Galveston, TX, USA; 2 Department of Biochemistry & Molecular Biology, Louisiana State University Health Sciences Center, New Orleans, LA CG present address, Depts. of Medicine and of Molecular & Cellular Biology, Baylor College of Medicine, Houston, TX, USA; 3 Department of Biology & Biochemistry, Centers for Biomedical & Environmental Genomics and/or Nuclear Receptors & Cell Signaling, University of Houston, Houston, TX, USA; 4 Department of Pharmacology & Toxicology, and Sealy Center for Structural Biology & Molecular Biophysics, Univ. of Texas Medical Branch, Galveston, TX, USA; 5 Present address St. Jude Children’s Hospital, Memphis, TN, USA

**Keywords:** 5 Aza-2’ deoxycytidine, Apoptosis, Dexamethasone, Epigenetic, Glucocorticoid, Glucocorticoid receptor, Methylation, p38, Phosphorylation, Leukemia, Lymphoid, Myeloma, High throughput sequencing, CEM, Molt-4, RPMI 8226

## Abstract

**Background:**

Glucocorticoids (GCs) are often included in the therapy of lymphoid malignancies because they kill several types of malignant lymphoid cells. GCs activate the glucocorticoid receptor (GR), to regulate a complex genetic network, culminating in apoptosis. Normal lymphoblasts and many lymphoid malignancies are sensitive to GC-driven apoptosis. Resistance to GCs can be a significant clinical problem, however, and correlates with resistance to several other major chemotherapeutic agents.

**Methods:**

We analyzed the effect of treatment with the cytosine analogue 5 aza-2’ deoxycytidine (AZA) on GC resistance in two acute lymphoblastic leukemia (T or pre-T ALL) cell lines- CEM and Molt-4- and a (B-cell) myeloma cell line, RPMI 8226. Methods employed included tissue culture, flow cytometry, and assays for clonogenicity, cytosine extension, immunochemical identification of proteins, and gene transactivation. High throughput DNA sequencing was used to confirm DNA methylation status.

**Conclusions:**

Treatment of these cells with AZA resulted in altered DNA methylation and restored GC-evoked apoptosis in all 3 cell lines. In CEM cells the altered epigenetic state resulted in site-specific phosphorylation of the GR, increased GR potency, and GC-driven induction of the GR from promoters that lie in CpG islands. In RPMI 8226 cells, expression of relevant coregulators of GR function was altered. Activation of p38 mitogen-activated protein kinase (MAPK), which is central to a feed-forward mechanism of site-specific GR phosphorylation and ultimately, apoptosis, occurred in all 3 cell lines. These data show that in certain malignant hematologic B- and T-cell types, epigenetically controlled GC resistance can be reversed by cell exposure to a compound that causes DNA demethylation. The results encourage studies of application to *in vivo* systems, looking towards eventual clinical applications.

## Background

Malignant cells may preempt the natural epigenetic mechanism of cytosine methylation to alter expression of genes so as to escape anti-cancer therapy. Epigenetics includes chemical modifications of DNA, and/or chromatin structural proteins such as nuclear histones, with resulting effects on gene transcription. Methylation of cytosine nucleotides in genomic CpG islands, often located in proximal promoter regions, is known to negatively affect transcription of certain genes. In cancer, disruption of the normal DNA methylation patterns can result in an aberrant epigenetic state with substantial consequences, such as repression of tumor suppressors, leaving cells susceptible to oncogenetic transformation [1-7]. The cellular epigenetic state can affect other factors as well, e. g. cell cycle arrest through p21^WAF1^ and activation of the p38 MAPK signal transduction pathway [8]. Expression of nuclear hormone receptors, including the GR, also has been shown to be affected at the epigenetic level [9-11]. Repression of gene expression based on DNA methylation can be reversed by analogs of cytosine such as 5 aza-2’ deoxycytidine (AZA), which incorporate into the genome of actively dividing cells and can render the cytosine nucleotide sites which they replace incapable of methylation [1,3,6,8,12]. Clinically, AZA alone shows promising activity against acute myeloid leukemia and myelodysplastic syndromes [1,3,12,13].

GC-driven apoptosis of several types of leukemic cells provides the rationale for use of GCs in many chemotherapy regimens. This GC action requires activation of the GR (nr3c1), a ligand-dependent transcription factor, with subsequent regulation of a complex gene network [14], one affected strongly by other signal transduction pathways, including those involving protein kinase A (PKA) and the mitogen-activated protein kinases (MAPKs) [15-18]. However, GC resistance can be a clinical problem, often associated with resistance to other drugs and poor overall prognosis. Examination of clinical leukemia specimens has revealed that resistance *in vivo* is occasionally due to mutation within the GR gene or loss of GR, but too often it is found that though the GR is present and unmutated, the receptor is ineffectual in causing apoptosis [19-22].

In 1983, it was discovered that in the mouse spontaneous thymic lymphoma cell line SAK8, the DNA methylation state could affect GC-sensitivity [23,24]. We subsequently tested AZA on dexamethasone (Dex)-resistant human leukemic CEM cells, producing a few sub-clones of apparent revertants to sensitivity [25]. Based on this preliminary work, we have now tested the hypothesis that apoptotic sensitivity to GCs in certain human hematologic malignancies is controlled via the epigenetic state of the genomic DNA by examining the ability of AZA treatment to restore GC sensitivity to cells from three hematological malignancies: two types of acute lymphoblastic leukemia (ALL), CEM clone C1-15 (preT or early T-cell), and uncloned MOLT-4 (T-cell), and a resistant myeloma cell line, RPMI 8226. We confirm that treatment with AZA can convert GC-resistant CEM cells to GC-sensitive and show that this effect extends as well to the other cell lines, representing both T- and B-lineage malignancies. After AZA treatment, some clones appear stably converted to GC- sensitive. We show that in this conversion, several cell line-specific effects relevant to GR function occur. These include altered GR expression from transcriptional start sites at specific untranslated exons located in CpG islands, hypomethylation, expression and sensitivity to GC of coregulatory factors that affect GR actions, and in the MAPK pathway, alterations known to be favorable for GR phosphorylation and action. Our present study connects the cellular DNA methylation state with the networked, hormone-driven apoptotic actions of the GR. The results encourage research at the *in vivo* level, and since AZA is already in clinical use for certain hematologic malignancies, our results open the possibility of extending use of demethylating compounds to revert GC resistant malignancies to GC sensitive.

## Results

### Brief exposure to the genomic DNA demethylating agent AZA restores the GC-dependent apoptotic response in each of three cell lines

**Figure 1:**
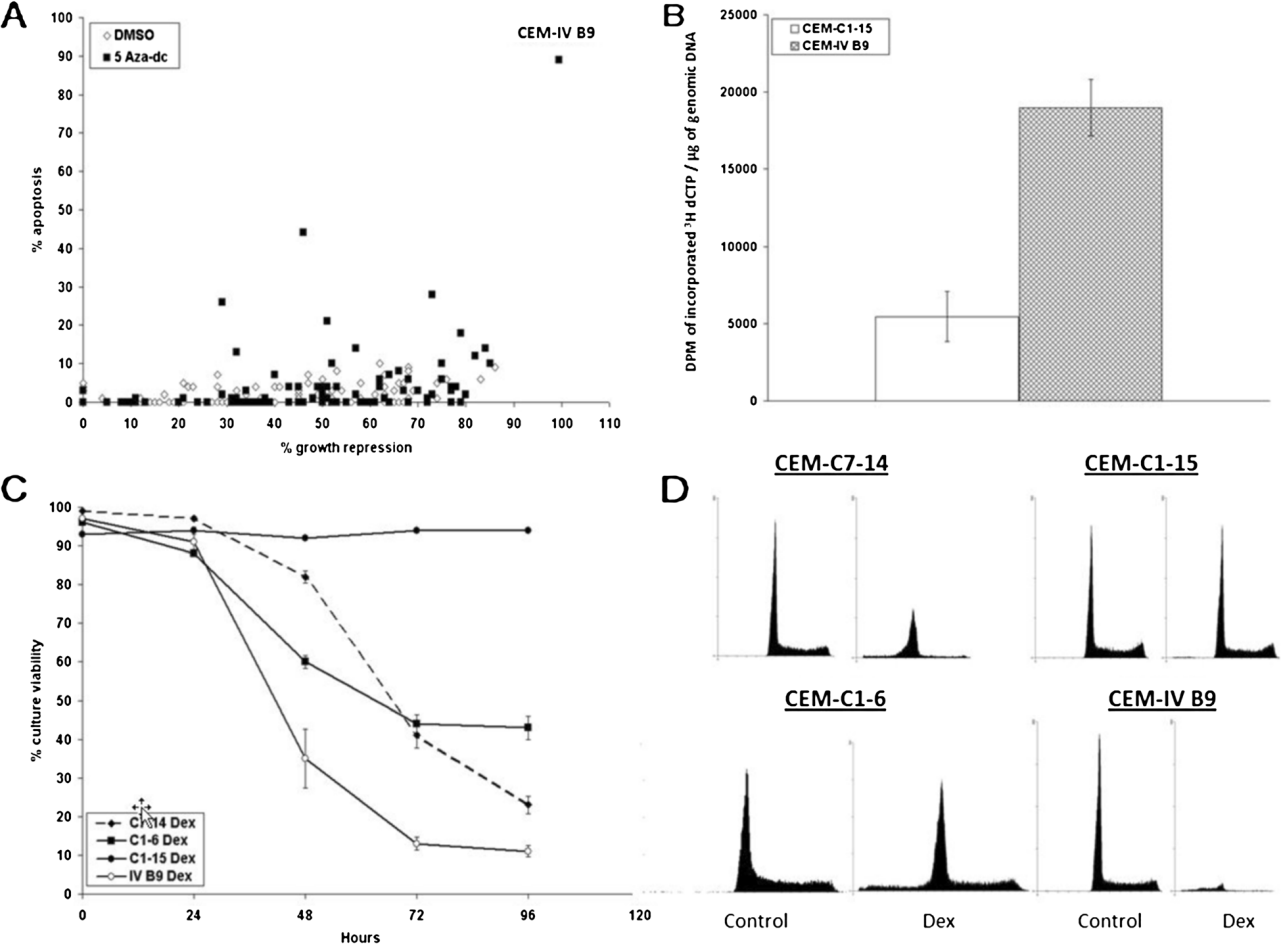
**AZA treatment of Dex**-**resistant C1**-**15 restores GC**-**dependent sensitivity to CEM clones. A**. CEM-C1-15 cells - treated with 100 nM AZA or DMSO for 48 hours were cloned by serial dilution to single cells and allowed to regrow. 105 DMSO and 94 AZA-treated outgrown clones were then exposed to 1 μM Dex or vehicle for 96 h and characterized for viable ( exclude trypan blue) and apoptotic (intact, trypan blue positive) cells. Plotted are % growth repression (total reduction in viable cells, x-axis) and % apoptotic cells within the remaining intact cell population (y-axis). **B**-**D**, Studies of revertant clone CEM-IV B9. **B**. Overall methylation of DNA from untreated CEM-C1-15 cells vs - clone CEM-IV B9, by cytosine extension assay- (DPMs of incorporated ^3^H dCTP per μg of genomic DNA). Error bars = 1 SD, n = 5. p = 0.0003. **C**. GC (Dex) sensitivity. Triplicate cultures of naturally Dex-sensitive CEM-C7-14 (dashed line, closed diamonds), Dex-resistant CEM-C1-15 (closed circles), and two - revertant clones: CEM-C1-6 (spontaneous revertant from C1-15, closed squares), and CEM IV B9 (demethylated revertant, open circles) were treated with 1 μM Dex and followed for 96 hours for numbers of viable cells. - Shown is the average percentage - of viable cells. Error bars = 1 SD -. **D**. Flow cytometry. The four - clones -of panel **C** were treated with vehicle (control) or 1 μM Dex for 96 hours and evaluated by flow cytometry. Data in graphs representative of biological n = 2. For IV B9 cells, extensive cell death and secondary, post apoptotic lysis allowed modelling of <20,000 events. All four panels are at same scale. Cell cycle results (avg. of 2 expts), see Table 1.

**Figure 2:**
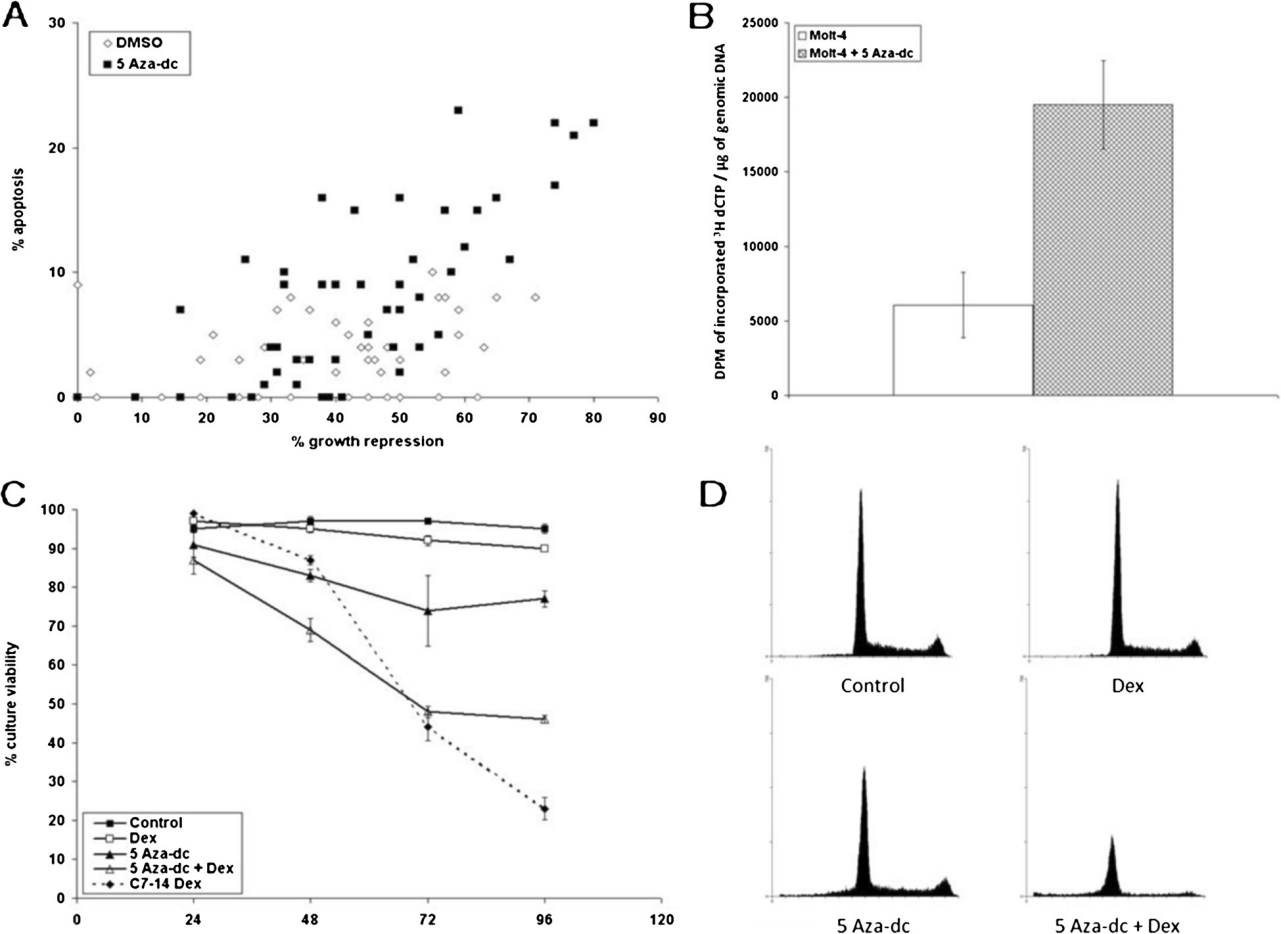
**Treatment with AZA restores sensitivity to GCs in Molt**-**4 cells. A**. Molt-4 cells treated for 48 hours with DMSO or 100 nM AZA were cloned by serial dilution to single cells and viable cell populations were allowed to regrow. % growth repression and residual apoptotic cells after 96 hours of 1 μM Dex treatment for control (49 clones, open diamonds) or AZA (49 clones, closed squares) are plotted. Upon further culture, the Dex-sensitivity of these clones proved to be unstable; therefore in **B**-**D**, experiments were performed on the MOLT-4 mass culture. **B**. DNA methylation of uncloned Molt-4 cells treated with DMSO vehicle or 100 nM AZA for 48 h. Data show DPMs of incorporated ^3^H dCTP per μg of genomic DNA. Error bars = 1 SD, n = 5. p = 0.001, DMSO vs. AZA **C**. Timing of response to Dex. Molt-4 cells were treated in triplicate with vehicle (control, closed squares), 1 μM Dex (open squares), 100 nM AZA (closed triangles), or 100 nM AZA for 24 hours before adding 1 μM Dex (open triangles). Average percentage of 3 independent replicates for cell culture viability following the addition of Dex. Sensitive clone CEM-C7-14 (C7-14, dashed line, closed triangles, data from Figure 1C) included for comparison. Error bars = 1 SD. **D**. Flow cytometry of uncloned MOLT-4 cells treated with AZA, Dex or AZA followed by Dex. Cells were treated as in **C**. with evaluation by PI staining and flow cytometry 72 hours after Dex. Each histogram (all histograms are same scale) results from the collection of 20,000 “events” where possible. n = 1. Extensive cell loss caused lower number of “events” in lower right hand histogram. Cytometric evaluations, Table 1.

**Figure 3:**
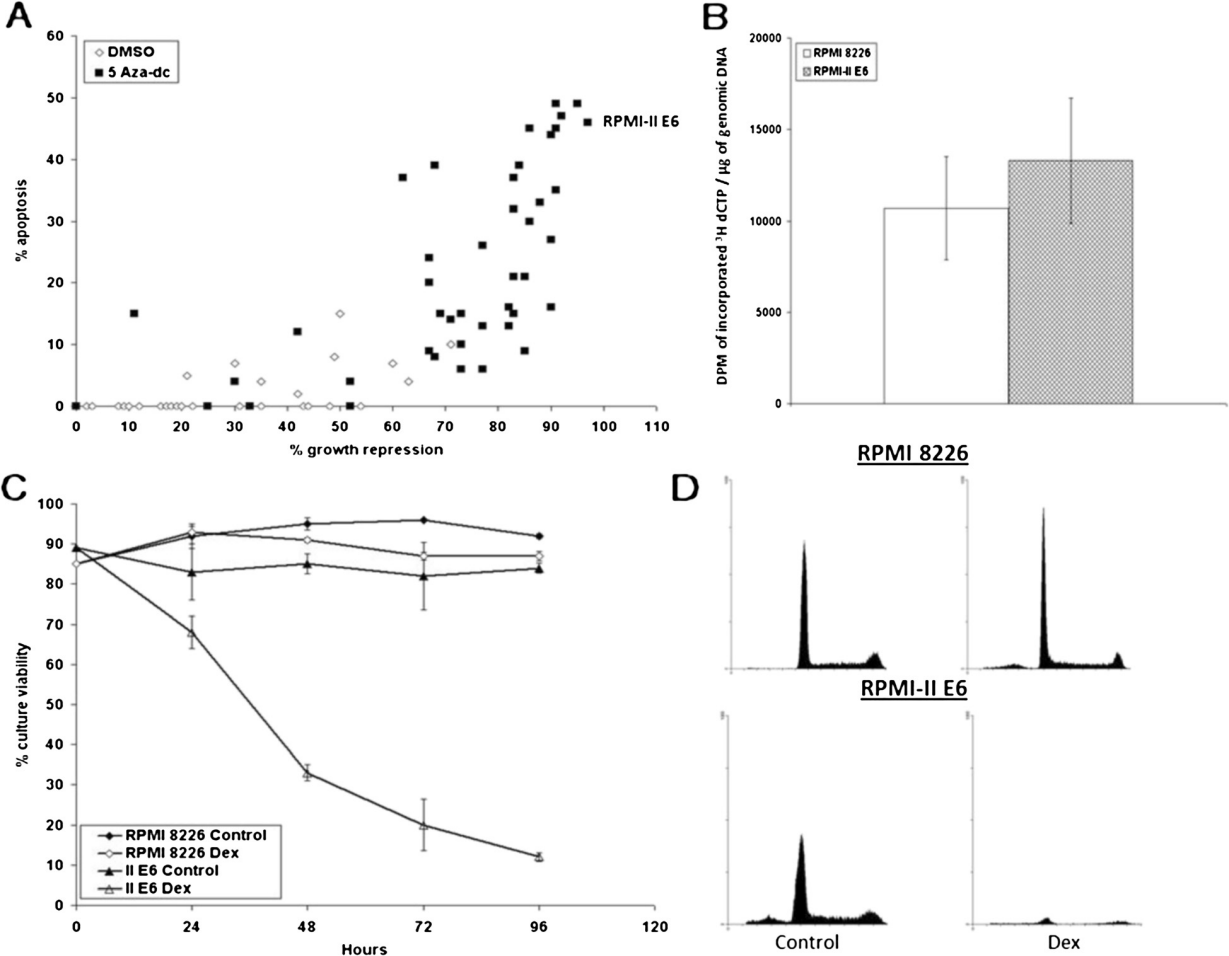
**AZA restores GC-dependent sensitivity to clones of multiple myeloma cells derived from the resistant RPMI 8226 parent. A**. RPMI 8226 cells were treated with 100 nM AZA or an equivalent volume of DMSO for 48 hours. Cells were then dilution cloned to single cells, and viable cell populations were allowed to regrow. Percentages of growth repression vs. apoptosis after 96 hours of 1 μM Dex treatment for control (50 clones, open diamonds) or AZA (47 clones, closed squares) are plotted as in Figure 1A. Revertant clone RPMI-II E6 is indicated. **B**. The methylation states of DNA for uncloned RPMI 8226 cells without AZA treatment and of revertant clone RPMI-II E6 were evaluated by cytosine extension assay. Data are presented as DPMs of incorporated ^3^H dCTP per μg of genomic DNA. Error bars = 1 standard deviation from the mean. n = 5 for RPMI-II E6 and 6 for RPMI 8226. p = 0.08, RPMI 8226 vs. RPMI-II E6. **C**. RPMI 8226 and II E6 cells were treated in triplicate with ethanol (control, closed diamond, closed triangles) or 1 μM Dex (Dex, open diamonds, open triangles) for 96 hours with final evaluation using Trypan blue exclusion to estimate vialble cells. Shown is the average percentage of 3 independent replicates for cells able to exclude the dye (% culture viability). Error bars = 1 standard deviation from the mean. **D**. RPMI 8228 and clone II E6 were treated with ethanol vehicle (control) or 1 μM Dex for 96 hours, then stained with PI and evaluated by flow cytometry. Each histogram represents the results from the collection of 20,000 events where possible. n = 3. Note that Y-axis scales are all identical; in the lower two panels, loss of cells due to lysis reduced the number of countable “events” in the cytometer. Also, see Table 1.

We evaluated the contribution of the epigenetic state to GC resistance in three commonly used model systems of human lymphoid hematologic malignancies: 1) CEM-C1-15, a GC-resistant clone of the pediatric ALL cell line CCRF-CEM; 2) Molt-4, an uncloned T-cell derived pediatric ALL cell line; and 3) RPMI 8226, an uncloned myeloma line (B-cell lineage). The cells of each system contain functional GR; yet each is highly resistant to GC-evoked apoptosis [26,27]. Initially, we treated each system with AZA for 24 h; then added Dex and followed the cultures over time. There was significant near-term restoration of sensitivity to Dex-driven apoptosis in each system. *Thus*, *the* % *reduction in live cells treated with AZA 24 h followed by Dex for 72 h*, *compared to 72 h Dex only*, *was 87*% *for MOLT*-*4* (*n* = *4*, *each in triplicate*), *74*% *for RPMI 8226* (*n* = *2*, *in triplicate*) *and 86*% *for CEM C1*-*15* (*n* = *3*, *in triplicate*). Since AZA incorporation into replicating DNA results in multiple phenotypes, possibly not entirely random [28,29], one could not expect every cell in the treated populations to convert to Dex sensitivity; so it is striking that in repeated experiments, such treatment always resulted in a near-term shift to sensitivity in a significant proportion of the population. In all three systems, phase-contrast visualization of the cells showed them to undergo the classic progression of apoptosis: cell shrinkage, nuclear condensation, karyorrhexis, and eventually, cell lysis. To estimate how many cells of each system were stably affected, we cloned each population after exposure to AZA or DMSO vehicle for 48 hours. About 1 month later, in a screening process, many clones were evaluated individually for Dex sensitivity. Because in some lymphoid cell systems Dex can arrest cell growth without causing apoptosis, we evaluated both repression of growth (total reduction in viable cells, and intact, yet apoptotic cells remaining at the end of the treatments. For this relatively high-throughput screen, we used trypan blue dye exclusion as a working definition of viability. Figures 1, 2 and 3 show results for CEM, MOLT-4 and RPMI 8226 cells, respectively. Panels 1A, 2A, and 3A show the data evaluating many individual clones as soon as clone outgrowth was sufficient for the assays. Each dot represents one clone, with % repression of growth on the x-axis, and % apoptotic (trypan blue positive) but still intact cells on the y-axis. While this analysis allows a rough estimate of the separation between growth retardation and ongoing apoptosis, it can underestimate the total quantity of cells lysed before the evaluations, since these are not “seen” by the instrument used. Nevertheless, the data in Figures 1A-3A show that in early clones from resistant cells pre-treated only with DMSO (open symbols), Dex caused varying degrees of growth repression with relatively few apoptotic cells to be found after 96 h. In contrast, in the clones derived from AZA treated cultures (closed symbols) Dex treatment often caused growth arrest plus a significant percentage of apoptotic cells at 96 h, suggesting ongoing apoptosis. Extensive growth arrest and Dex-dependent apoptosis generally were correlated in cells from all three lines pre-treated with AZA, consistent with our previous observations on the cell-cycle effects of Dex on sensitive CEM cells [30]. We conclude that the AZA effect is widespread, due to effects on many individual cells, and that the acute sensitization to Dex may sometimes persist.

*CEM cells* (Figure 1). Visual and Vi-cell evaluation of the CEM-C1-15 cell clones indicated that no clone in this experiment treated with DMSO alone reverted to GC-mediated apoptotic sensitivity. On the other hand, many clones from AZA-treated C1-15 cells showed high Dex sensitivity. Clone IV B9 (extreme upper right, Figure 1A) derived after AZA, showed a striking reversion to Dex-sensitivity, and it remained phenotypically stable over 4 months in continuous culture and after repeated freeze-thawing; therefore it was taken for further evaluation. Cytosine extension assay results (Figure 1B) indicated that clone IV B9 DNA was 4-fold hypomethylated relative to CEM-C1-15 cells. High throughput DNA sequencing showed regions of reduced methylation in DNA in all autosomes, relative to the resistant CEM C1-15 cells. From CEM C1-15, CEM C7-14 and revertant IV B9 cells, non-methylated and methylated DNA were separated and sequenced (Methods). The average coverages for non-overlapping 5,000 bp windows were calculated across all chromosomes, examining all DNA and also only unique gene locations. Coverage comparisons of C1-15 and C7-14 sequences enriched for methylated DNA showed that many C1-15 sequences contained regions- both repeat sequences and unique sequences- with high methylation that were not found in the C7-14 data. Similarly, a plot of sensitive revertant IV B9 against its resistant parent C1-15 revealed loss of methylation at many sites in IV B9.

Comparisons of Dex-sensitivity between clone IV B9, natively sensitive CEM-C7-14, the spontaneous revertant CEM-C1-6 (described in [27]), and the resistant parental clone CEM-C1-15, showed IV B9 to be most Dex-responsive in both loss of viable cells and in rapidity of that response (Figure 1C). Clone IV B9 also was sensitive to lower concentrations of Dex than other sensitive clones, shown by the leftward shift of the dose response curve and a greater lytic response to Dex (Figure 1C, 1D, Table 1). Flow-cytometric evaluation of propidium iodide (PI) stained DNA in cells reinforced these data (Figure 1D, Table 1).

**Table 1:** **Cell cycle analysis, Figures**1, 2 and 3**D**

**Cell type**	**Treatment**	**% G1**	**% G2**	**% S**	**% A**	**% Deb**
**Figure**1**D, CEM clones**						
**C7-14**	**Control**	**57.5**	**6.125**	**36.27**	**00.035**	**00.845**
**C7-14**	**Dex**	**75.87**	**29.45**	**21.19**	**34.18**	**25.92**
**C1-6**	**Control**	**50.99**	**4.18**	**44.83**	**00.075**	**1.715**
**C1-6**	**Dex**	**64.24**	**7.31**	**28.44**	**00.02**	**25.43**
**C1-15**	**Control**	**51.695**	**8.705**	**39.6**	**00.005**	**00.88**
**C1-15**	**Dex**	**52.815**	**9.355**	**37**,**8**	**2.07**	**2.055**
**IV-B9**	**Control**	**58.415**	**5.975**	**35.6**	**00.09**	**1.405**
**IV-B9**	**Dex**	**58.615**	**5.435**	**35.95**	**58.855**	**18.75**
**Figure**2**D, MOLT-4 (uncloned)**	**Control**	**58.82**	**8.57**	**32.62**	**00.03**	**5.83**
	**Dex**	**62.85**	**8.14**	**29.0**	**00.12**	**2.94**
	**AZA**	**58.02**	**10.00**	**31.8**	**00.66**	**21.3**
	**AZA +** **Dex**	**73.64**	**6.64**	**19.72**	**00.00**	**34.91**
**Figure**3**D, RPMI (uncloned)**	**Control**	**61.71**	**11.73**	**26.46**	**00.00**	**2.32**
	**Dex**	**59.88**	**9.31**	**30.81**	**6.40**	**2.87**
**RPMI clone II E6**	**Control**	**56.02**	**11.51**	**32.47**	**6.72**	**11.30**
	**Dex**	**51.31**	**42.6**	**6.09**	**1.5**	**46.58**

*MOLT*-*4 cells*. Of the 3 cell systems, MOLT-4 showed the greatest acute response as a mass culture (Figure 2 and data cited in text). Clearly, there also is a shift to more GC response in the demethylated clones from MOLT-4 cells (Figure 2A). Upon extended culture, the Dex-sensitive phenotype of these clones proved to be unstable, however. The data in Figure 2B-2D therefore evaluate the Molt-4 mass culture following acute treatment with AZA and Dex. Test of the DNA after 48 hours of exposure to AZA confirmed demethylation (Figure 2B). Treatment of the cells with AZA for 24 hours followed by the addition of Dex resulted in a marked loss of cell viability over time (Figure 2C). After treatment with AZA only, there was a 20-25% reduction in viable cells (% culture viability, y-axis) Dex alone reduced viability by only about 5%. AZA followed by Dex, however, reduced viability in this experiment by 50%. These results support the conclusion that an epigenetic state is responsible, at least in part, for the GC resistance of many Molt-4 cells, though the AZA/Dex synergy could include additional mechanisms. Flow cytometry also indicated that the mass culture of MOLT-4 cells treated with AZA had undergone some cell loss (Figure 2D lower left panel and Table 1) However, there was a drastic reduction of intact cells after treatment with AZA followed by Dex, and an increase of cell debris, coupled with a reduction in total viable cells, resulting in a reduction in cells available for evaluation by flow cytometry. (Figure 2D, lower right panel. The scale for all histograms in Figure 2D is identical.) The cytometric evaluation also showed a shift of the residual population into G1, typical of the apoptotic effect of Dex in many lymphoid cell systems, e.g. CEM [30]. Since phase-contrast microscopy had shown these cells to go through the morphologic phases of apoptosis, we conclude that at this 96 hr time point, many cells in the culture had gone on to secondary necrosis/lysis.

*RPMI cells* (Figure 3) The mass culture of RPMI 8226 myeloma cells also became sensitive to Dex-induced lysis after treatment with AZA. Many outgrown clones showed significant growth arrest and ongoing apoptosis (Figure 3A). The most sensitive clone (II E6) was stable in phenotype and was kept for further analysis. In this clone, cytosine extension assay did not detect a statistically significant shift in total DNA methylation (Figure 3B). Exposure of clone II E6 to Dex revealed a significant loss of viable cells by 48 hr, comparable to CEM clone IV B9, and faster than CEM clones C1-6 and C7-14 (Compare Figures 1C and 3C). Flow cytometry also showed that under control conditions, clone II E6 sustained an ongoing low level of apoptosis, presumably a result of the earlier AZA treatment (lower left panel, Figure 3D and Table 1) After 96 hr in Dex, clone II E6 showed a near complete loss of viable cells, and only a few remaining cells had sub-diploid DNA content (Figure 3D Note, the scale of the Y-axis in all panels of 3D is identical, and see Table 1).

### Clonogenicity is lost in RPMI 8226 cells treated with AZA and Dex

Because the % apoptosis appeared low in the method used for evaluation of RPMI 8226 cells, we performed a clonogenicity assay, a classic measure of cellular malignant potential- the ability to restore a population from single cells. To test the effect of altered methylation on the clonogenicity of RPMI 8226 cells, we treated with vehicle alone, Dex, AZA, or AZA plus Dex, and subsequently cloned each sample by serial dilution in fresh medium in multi-well plates. After seeding on average, 1000, 100, 10, and 1 cells per well, two weeks were allowed for growth, and viable cells were counted by Trypan blue exclusion assay. All the wells seeded initially at 1000 were too overgrown to count and were not evaluated further. Dex treatment alone had, if any, slight positive effects on cloning efficiency and the ability of the cells to repopulate from any starting density. Alone, AZA reduced the number of cells grown from 100 , 10, or single cell wells but failed to block clone formation. Combination of AZA and Dex resulted in a complete loss of clonogenicity for single cells and a marked reduction in growth in the 10-cell wells (Table 2). Thus, combination treatment with AZA and Dex significantly compromised the clonogenicity/oncologic potential of RPMI 8226 cells.

**Table 2:** Clonogenicity of RPMI 8226 cells

**Starting cells/well**	**10**	**1**	**10**	**1**
	Cloning efficiency		Viable cells/well	
	(% wells w viable cells)		(×10^-5^) ± SD	
Control	100	50	6.0 ± 1.3	0.15 ± 0.3
Dex	100	75	6.9 ± 1.3	1.2 ± 1.5
AZA	100	50	0.7 ± 0.6	0.06 ± 0,1
AZA + Dex	75	**0**	**0.14 ±** **0.1**	**0**

Clonogenicity of RPMI 8226 cells is reduced by treatment with AZA followed by Dex. RPMI 8226 cells were incubated with 100 nM AZA or DMSO vehicle for 40 hours before addition of ethanol vehicle (control) or 1 μM Dex for an additional 96 hours. Cells were then serially diluted to initial staring densities of 100, 10, or single cells in cloning medium. Cells from independent wells for each treatment were counted using a Trypan blue exclusion assay 2 weeks after cloning. Shown are cloning efficiency, range and sum of viable cells from all wells seeded with 10 or single cells for each treatment. Data in bold emphasize the AZA+Dex effect.

*The data of Figures*1*,*2*and*3*and Tables*1*and*2*demonstrate that in three cell systems, AZA treatment restores sensitivity to Dex-induced cell death. In an initial exploration of mechanisms, we examined the effects of the AZA treatment on some known important elements in Dex-dependent apoptotic pathways.*

### GC-stimulated events involving p38 MAPK , a feed*-*forward stimulator of GR action*,* are important for glucocorticoid-dependent apoptosis in all 3 cell systems

**Figure 4:**
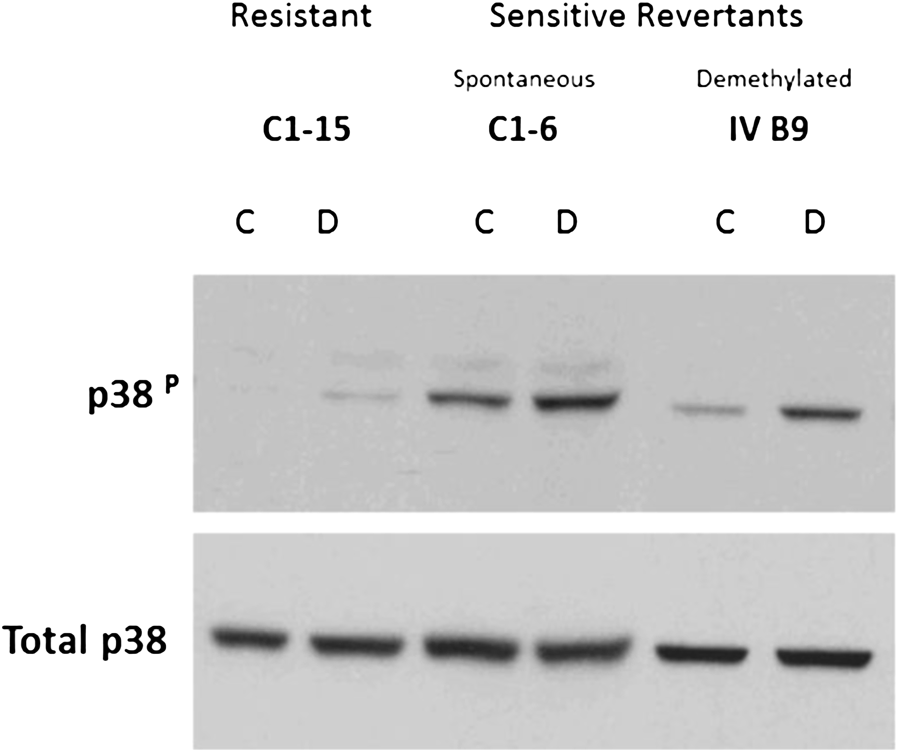
**Dex**-**stimulated p38 MAPK phosphorylation**/**activation is increased in revertant CEM clones.** CEM-C1-15 (C1-15 resistant), CEM-C1-6 (C1-6 spontaneous sensitive revertant), and CEM-IV B9 (IV B9 hypomethylated sensitive revertant) were treated with ethanol vehicle (C) or 1 μM Dex (D) for 16 hours after which time whole cell lysates were analyzed using immunoblots with an antibody specific to phospho-p38 protein. Membranes were subsequently re-probed with an antibody recognizing total p38 protein. Illustrated is a representative blot of 4 independent experiments for IV B9 cells, all with similar results. C1-15 and C1-6 are included for comparison n = 4. We have previously published detailed analysis of the MAPK pathway in these CEM clones [17].

**Figure 5:**
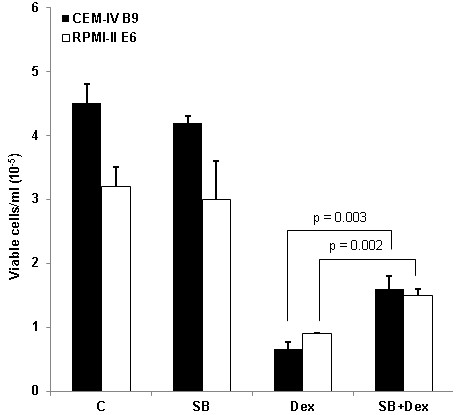
**Pharmacological block of p38 MAPK partially inhibits Dex**-**dependent cell death in CEM and RPMI clones converted to Dex**-**sensitive by AZA treatment.** CEM IV B9 and RPMI II E6 cells were diluted to a starting density of 1×10^5^ viable cells/ml, pre-treated for 1 hr with ethanol/DMSO vehicle (control) or 1 μg/ml p38 inhibitor SB203580 (SB) for CEM cells or 2 μg/ml SB for RPMI cells, and subsequently exposed to 1 μM Dex alone (Dex) or the combination of SB plus Dex (SB + Dex) for an additional 48 hours. Viable cells were counted using an automated cell counter. Depicted are the average results and standard deviations from 3 independent experiments each consisting of triplicate replicates for each treatment. Paired t-tests were performed on viable cell numbers and culture viability for (Dex) vs. (SB + Dex). n = 3.

GC-stimulated p38 MAPK activation plays a major role in the glucocorticoid-dependent apoptotic mechanism in a variety of malignant lymphoid cell lines, including CEM, Molt-4 and RPMI 8226 [17,18,27,31-33]. Elevated p38 relative to JNK and ERK MAPKs favors an apoptotic response in a variety of malignant lymphoid cells [32]. The GR is phosphorylated by p38 (and other kinases) at ser 211, which enhances GR transcriptional and apoptotic activities [17,18,27,33]. Comparisons of the resistant parent CEM-C1-15, its demethylated clone IV B9, and the spontaneous revertant CEM-C1-6 revealed a strong increase in phosphorylated (i.e., activated) p38 MAPK in response to Dex stimulus in both IV B9 and C1-6 cells, and at best, a very slight increase in C1-15 cells. (Figure 4). Levels of total p38 MAPK protein remained unchanged, consistent with earlier reports [17,18]. To test the importance of the GC-stimulated p38 activity for apoptosis, the demethylated, stable revertants CEM IV B9 and RPMI II E6 were treated with the p38 inhibitor SB203580 for 1 hour before the addition of 1 μM Dex, and viable cells were enumerated 48 h later.With the SB added to block p38, there was statistically significant protection from Dex-driven apoptosis for the SB-treated cells in both systems, with an increase in total viable cells after Dex (Figure 5). In the SB-treated cultures, there was also a statistically significant increase in the % of viable cells in the cell populations still present at the end of the experiment. This fraction increased from 33% (Dex only) to 62% (SB + Dex) (p = 0.001) for IV B9 cells, and in II E6 cells, from 48% to 68% (p = 0.003). These results are consistent with partial inhibition of the Dex-induced ongoing apoptosis. Also, in Molt-4 cells, the uncloned, mass culture system, treated acutely with AZA, followed by Dex with or without SB, modest, but statistically significant protection was seen when the p38 blocker was added: AZA alone reduced culture viability by 25%; AZA followed by Dex reduced viability by 60%. There was a statistically significant 30% gain in viable cells when SB was added to the Dex + AZA treatment: p = 0.02, n = 3. This partial inhibition of cell death in all three systems is as expected, since ser211 phosphorylation of the GR enhances, but is not essential for GR actions. *The data above identify p38 as one possible AZA*-*sensitive node shared by all three cell systems in the known complex of pathways initiated by Dex that lead to apoptotic cell death. GR is known to affect p38 function and vice versa. We therefore further examined aspects of GR function. Both sufficient quantity and activity of the GR are known to be essential for it to drive lymphoid cell apoptosis*.

### GR functions are enhanced by differing mechanisms in CEM and RPMI 8226 cells

**Figure 6:**
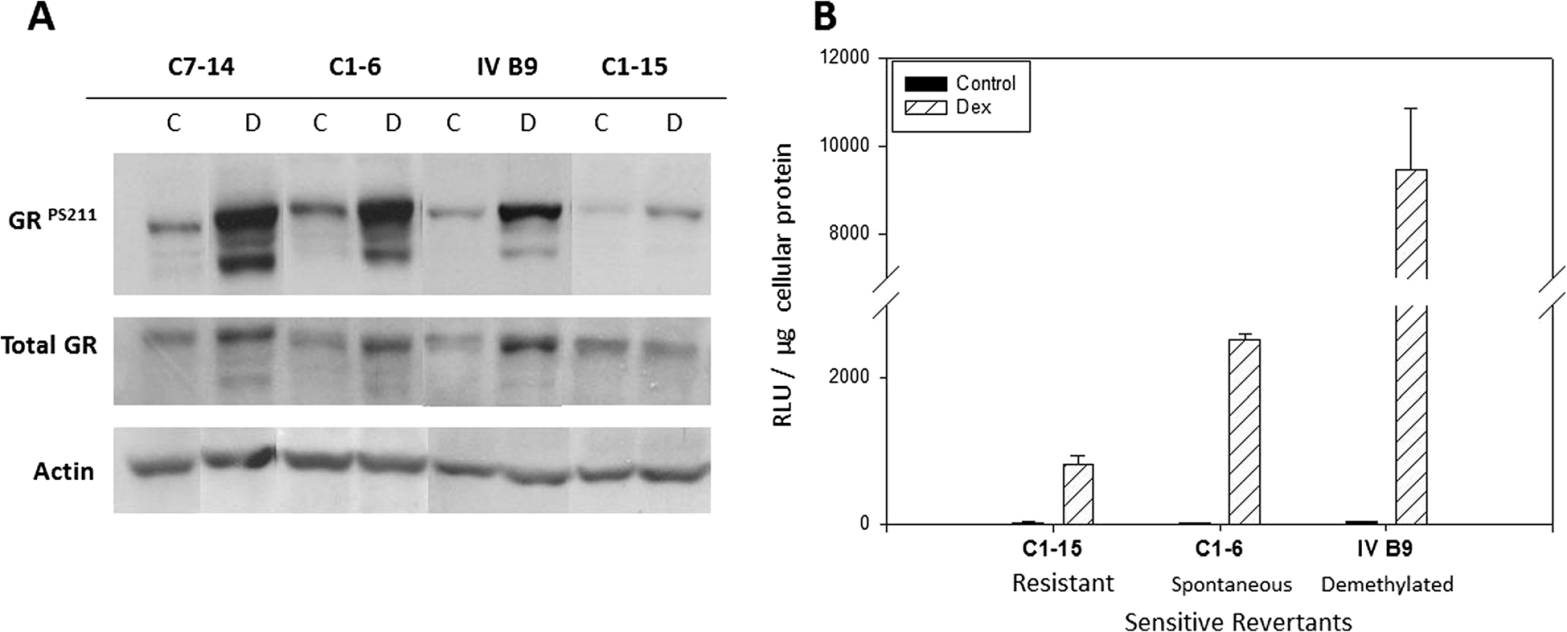
**GR site**-**specific phosphorylation**, **auto**-**induction**, **and potency in Dex**-**sensitive and** –**resistant CEM cells. A**. GR ser211 phosphorylation and auto-induction. Naturally Dex-sensitive CEM-C7-14 (C7-14), Dex-resistant CEM-C1-15 (C1-15), and two resistant-to-sensitive revertant clones; CEM-C1-6 (spontaneous revertant, C1-6), and CEM-IV B9 (demethylated revertant, IV B9) were treated in triplicate for 16 hours with 100 nM Dex (D) or an equivalent volume of ethanol vehicle (C). Cell lysates were then analyzed for phospho-GR S211, total GR, and actin by immunoblot. Depicted is a representative blot from 3 independent samples done, all with nearly identical results. n = 3. The data shown are from a single gel, with irrelevant sections removed, for clarity. **B**. Dex/GR-dependent transcriptional activity. CEM; C1-15, C1-6, and IV B9 cells were transfected by electroporation with a GRE- dependent luciferase reporter plasmid. Cells were then divided into triplicate replicates and exposed to either ethanol vehicle (control) or 1 μM Dex for 24 hours after which time extracts were made and luciferase activity analyzed. A representative experiment is shown of average RLUs normalized to μg of protein in the cellular lysate. Error bars = 1 standard deviation from the mean. Total biological replicates, n = 2 for C1-15; 5 for C1-6; and 7 for IV B9.

The p38 effects suggest one node of common action, highly relevant to GR activity, in the AZA-reverted cells of all three systems. However, considering the complex networks of interacting cellular signal systems, it is not surprising that additional, cell system-specific, non-overlapping effects influencing GR levels and activity were found. *In the CEM cell system* we observed: enhanced GR function, induction of GR protein and GR phosphorylation, and use of GR alternative promoters. Figure 6A shows that Dex induced GR protein in naturally sensitive clone C7-14 cells, in C1-6, the spontaneous revertant to sensitivity from C1-15 cells, and in the revertant clone IV-B9, obtained from C1-15 cells after AZA treatment. Dex did not induce GR protein in C1-15 cells. In C1-15 cells, there was lower basal GR ser211 phosphorylation and only weak induction, compared to the other three, Dex-sensitive cell clones. GR-driven transcriptional activity was consistent with these results. Cells were transfected with a GRE-driven luciferase reporter construct (Methods) and treated with Dex. There was highly increased GR transcriptional activity in both revertant clones C1-6 and IV B9 compared to C1-15 (Figure 6B). Revertant IV B9 showed at least 2-fold elevated activity over that of the spontaneous revertant C1-6, consistent with the cell death and GC dose-response data in Figure 1. It appears that the phosphorylation state of the GR at S211, total GR protein levels, and transactivation potential can be controlled by epigenetic mechanisms in this system.

**Figure 7:**
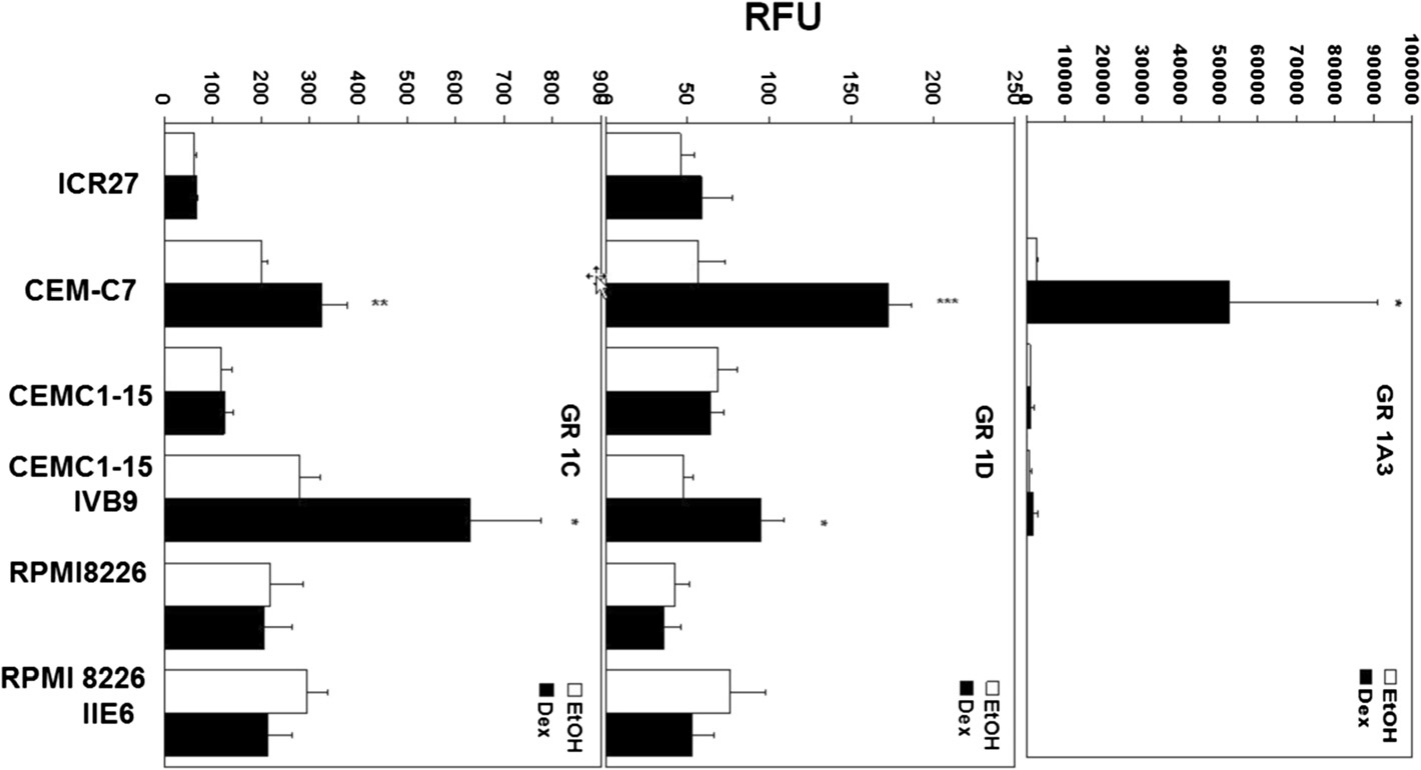
**Levels of transcription from three important GR untranslated first exons.** Shown are expression levels of transcripts from, top to bottom, GR exons 1A3, 1D, and 1C, expressed in relative fluorescence units (RFU). RNA was quantified using real-time PCR as outlined in Materials and Methods. The cell line or clone being tested is indicated along the abscissa. White bars, treatment with ethanol vehicle; black bars, with Dex.

Considering the Dex-dependent auto-induction of GR in these cells, and the correlation between such induction and apoptosis that has been reported [34], we evaluated the promoter-specific, basal and Dex-driven expression of the GR in revertant clone IV B9, compared to other relevant CEM clones. There are 11 untranslated exon 1 sequences at the 5’ end of the human GR [35], arising from specific human GR gene promoters. The promoter (s) for transcripts 1A and 1I are ~25 kbp upstream of exon 2, the first peptide coding exon and do not lie in a CpG island. The remaining promoters are proximal, located in the GC rich ~5 kb directly upstream from Exon 2 [36]. Hypothesizing that expression from 1A would be unaffected by AZA treatment, while transcription from one or more of the proximal promoters would be increased, we measured transcripts from GR exon 1A3, a T-cell selective promoter, and from exons 1D and 1C, two of the GR’s principal promoters in the proximal CpG-rich zone. Though transcription from promoter 1A3 is strongly induced in CEM C7 cells, this promoter is neither induced nor even expressed well in spontaneously resistant CEM clones ICR 27, C1-15, or in sensitive IV B9 cells. Dex-dependent induction of transcription did not occur from GR promoters 1C and 1D in resistant C1-15 cells, or in a spontaneously resistant CEM clone, ICR 27. This induction was seen, however, in the Dex-sensitive C1-15 revertant IV B9 (Figure 7). Limited pyrosequencing experiments could not confirm altered methylation of the proximal GR promoter. Pyrosequencing of a few short regions of the GR proximal promoter region showed a tendency for greater methylation at one or two positions in CEM C1-15 cells as compared to C7-14 cells (data not shown); however. pyrosequencing of the full 5000 bp promoter was beyond the resources available for this study. It will require further experiments in the future to clarify whether the GR promoter methylation state directly accounts for the shift in promoter usage or whether this is an effect secondary to alterations elsewhere in the genome.

### RPMI 8226 myeloma cells: The GC-sensitive revertant II E6 has altered expression of several genes known to affect GR-driven transcription

**Figure 8:**
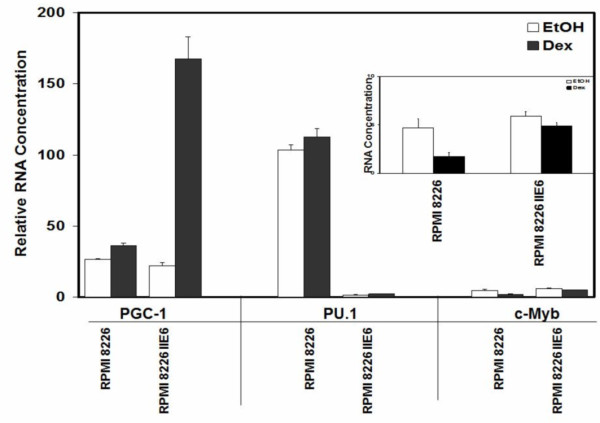
**Expression and regulation of three modulators of GR actions in IIE6**, **a Dex**-**sensitive revertant clone from RPMI 8226 cells.** RNA was quantified using real-time PCR as described in d Methods. Shown are the RNA expression levels, before and after Dex treatment, for GR CoActivators PGC-1a and c-Myb, and the GR CoRepressor PU.1. The inset shows the c-Myb data on an expanded ordinate.

We also compared expression from three GR promoters in Dex resistant RPMI 8226 cells and their stably sensitive clone II E6. Transcription from promoter 1A3 was virtually nil, whereas there clearly was transcription from 1D and 1C (Figure 7). None of the promoters was induced by Dex in these cells. However, other mechanisms known to bolster GR actions were altered, viz. the expression levels and GC inducibility of the genes for 3 of 5 coregulatory proteins known to affect GC actions in lymphoid cells [37-39]. Two, c-Myb and PGC-1a (PGC-1 alpha); act as GR coactivators; two as corepressors- PU.1 and Tel. Spi-B, the fifth coregulator, seems to interact with GR function, but without a clearly defined role as yet. None of these showed a clear pattern that seemed relevant to the CEM revertant (data not shown), but in the RPMI 8226 cell system, three of the five were altered so as to enhance GR activity (Figure 8). Similar basal levels of coactivator c-Myb were found in RPMI 8226 cells and its sensitive revertant II E6, but expression was significantly reduced by Dex only in the resistant 8226 cells (Figure 8 right hand bar graphs and inset). Coactivator PGC-1a also showed similar basal levels in parent and revertant but was strongly induced by Dex in the sensitive revertant. Corepressor PU.1 did not respond to Dex, but its level in the sensitive revertant cells was only about 1% of that in the resistant RPMI 8226 cells. Tel and Sp1B levels did not correlate with cellular response to Dex. Interestingly, the regulation of coactivators and corepressors plays a role in GR action in the pre-B-ALL 697 cell line [38,39]. Thus, in two cell lines in the B-cell lineage (RPMI 8226 and 697) the regulation of coactivators and corepressors is important for GC action. *In sum*, *expression levels and*/*or Dex regulation of three genes coregulatory for GR actions are altered in the Dex*-*sensitive RPMI 8226 revertant clone in a manner consistent with restoration of Dex sensitivity through enhanced GR actions*.

## Discussion

The central finding of the research reported here is that treatment of three different lymphoid cell lines with AZA converted the cells from glucocorticoid-resistant to glucocorticoid-sensitive. The data show that the GC Dex, given to each resistant cell system soon after AZA, caused the death of the large majority of cells in the cultures (Data in Results text and Figures 1, 2 and 3). Consequent important issues are, what is the nature of the cell death and by what mechanisms is it achieved? As to the nature of the cell death, both the knowledge that GCs cause lymphoid cell apoptosis and our data herein suggest that at least the major death pathway in the AZA, then Dex-treated cells is indeed apoptosis. The original discovery of apoptosis was made by histopathology [40,41], and indeed, lymphoid cells are one of the classic systems used to describe the phenomenon . Following the recommendations of a large panel of cell death experts [42], we employed several methods for evaluating the process- phase contrast microscopy, dye exclusion, cell shrinkage, and cell cycle analysis- and the results all point to apoptosis. It may be useful in the future for ourselves or others to examine additional elements of apoptosis pathways, of interest to particular labs.

As to mechanism, the first question is whether altered DNA methylation is involved. AZA is well-known to cause reversal of DNA methylation, but the drug can also have other effects. We strongly suspect that a major reason for the restoration to GC sensitivity is altered expression of genes suppressed by DNA methylation in the resistant cells, but we cannot rule out the possibility that some of the restored sensitivity in the short-term is due to a combination of GC and other AZA effects. These seem much less likely in long-term revertant cell clones. In our previous work, comparisons of gene expression between Dex-sensitive clone CEM-C7-14, Dex-resistant CEM-C1-15, and the spontaneous resistant-to-sensitive revertant CEM-C1-6 [27] had suggested that GC-resistance in CEM cells could be due to altered regulation of large gene networks, a result consistent with epigenetic control. That is, the extent and pattern of gene expression in the resistant cells and their sister sensitive cells seemed too broadly different to be due to mutation of a single gene [27]. Also, our previous preliminary results on the role of DNA methylation in regulating GC-driven apoptosis supported this interpretation [25]. Consequently, we tested the hypothesis that DNA methylation of hematological cells could result in loss of the GC-sensitive phenotype. We treated with AZA to try to alter the methylation state of 3 cell systems from different GR-positive, GC-resistant human hematological malignancies: acute lymphoblastic leukemias Molt-4 (T-cell), and CEM clone C1-15 (CD4+/CD8+) and the multiple myeloma (B-cell) line RPMI 8226. (CEM cells have been designated as either T or pre-T, because they express surface antigens for both CD4 and CD8, with CD4 predominating quantitatively). Exposure of these B- and T-lineage lines to AZA results in sensitization of their mass cultures to Dex-evoked apoptosis, measured by Dex-dependent: reduction of viable cells, increased apoptotic, still intact cells, followed by eventual cell lysis, and by loss of clonogenicity. We therefore conclude that most of the cell death seen in the revertant cells is apoptotic, given the known pro-apoptotic effects of GCs in lymphoid cells. Total cell DNA methylation was reduced in the CEM and MOLT-4 cells, but not in the revertant RPMI clone. Of course, a reduction in overall methylation is by no means necessary for demethylation at critical sites to have taken place. Considering that these cells had gone through many divisions, and the known demethylating effect of AZA, we conclude that our data best support the interpretation that apoptotic sensitivity to GCs in cells from certain lymphoid malignancies can be controlled by the epigenetic state of the DNA, as our previous work suggested. We note that epigenetic control of GC sensitivity at the histone methylation level has also been reported [19,43]. The importance of epigenetic regulation in hematologic malignancies is well documented, and efforts are being made to to manipulate such regulation in vivo [44-51]. Therapy with AZA-like drugs directed at myelodisplastic syndrome and AML has given promising results.

As to the mechanisms responsible for the switch to GC sensitivity, and considering the complex regulatory networks that control cell health and death and the widespread alterations in DNA methylation, no doubt multiple systems will have been affected. We therefore made choices as to initial studies of mechanism. Because the reversal of phenotype proved to be unstable in MOLT-4 clones, we limited experiments to comparing the effects of AZA, Dex and AZA followed by Dex on the mass cultures (Figure 2 and Results, text). While this limits detailed studies of the detailed pathway mechanisms, due to population heterogeneity, it does allow examination of the effects of each treatment on the system. The data show that AZA alone had an acute toxic effect, Dex alone had little, and the sequential combination was much more effective in causing cell death. Stable clones could be obtained from the CEM and RPMI cells. We elected to pick the most clearly sensitive of these for more detailed work-up. While one cannot expect to discover the mechanisms that occur in every cell or in vivo from such studies, one can hope to find pathways of importance. Such an approach has proven valuable innumerable times in the history of science. As a first, obvious system to investigate, we explored the GR and several systems that control its actions in AZA-treated, stable clones from the CEM and RPMI cells. The known apoptotic effects of GCs in lymphoid cells require adequate levels of functional GR. We document several processes that enhance the activity of the GR, in a cell-line specific manner. In the stably sensitized clone of CEM cells, we noted restoration of several relevant GR properties to levels found in GC-sensitive cells: transcription from important GR promoter sites; GC-mediated GR induction; and site-specific phosphorylation of the GR, coupled with an increase in its transcription-factor potency.

In the RPMI 8226 system, reversion to GC sensitivity did not correlate with increased GR autoinduction or quantity. Instead, it correlated with alterations in expression of GR co-regulators c-Myb, PGC-1a and PU.1 in ways that, according to published reports of their actions [38,39,52], should enhance GR activity. Detailed follow-up of this result will require experiments beyond the scope of this paper.

Common to all three model cell systems was activation of p38 MAPK; a kinase that phosphorylates and thus activates the GR and is important for GC-driven apoptosis of several types of malignant lymphoid cells [32]. We showed that the balance between MAP kinases JNK, ERK and p38 is one determinant of GC sensitivity [17]. This relationship was shown to be important for GC sensitivity in 8 unrelated hematologic cell lines [32]. We hope that these initial findings as to mechanism will lead to experiments in other lymphoid systems, and that the results herein will have applicability beyond the cell lines we tested.

Our results may have potential for eventual clinical relevance. GCs are used widely in the treatment of lymphoid malignancies, due to GC-dependent apoptosis of the malignant cells. But resistance can occur, and resistance to GC often correlates with resistance to other chemotherapeutic drugs. Though simple loss of GR has been documented in lymphoblasts from late-stage resistant ALL, and from some lymphomas, this mechanism does not seem to explain the majority of clinical resistance to GCs [20,21,53]. That the GR is required for GC-driven apoptosis, however, seems quite clear. The GR is one hub of a complex set of interactive networks that include at least: c-myc, the MAPKs, PKA, polyamines, and redox pathways [31,32,40,54-58]. Thus it is encouraging that despite this complexity, treatment with AZA resulted in sensitization to GC-induced apoptosis of 3 differing lymphoid cell lines. Thus, the data presented herein provide encouragement for additional studies aimed at eventually translating DNA demethylation to clinical use in lymphoid malignancies, as a means of producing/enhancing sensitivity to GC.

## Conclusions

1. Short-term treatment with AZA produced acute- and in some clones, prolonged sensitivity to GC-driven apoptosis in 3 lines of malignant lymphoid cells (2 T cell or pre-T and one of B cell lineage).

2. One nodal function seen to be altered in all three systems was the activation of p38 MAPK, a known GR-driven forward feedback system important for eventual lymphoid cell apoptosis.

3. Further mechanisms were cell-line specific and included alteration in use of GR promoters, GC-driven induction of the receptor, and altered regulation of cofactors that modulate GR function.

4. Given the successful use of DNA demethylating agents in vivo for the treatment of certain malignancies, these results encourage further trials of such agents to restore/enhance GC sensitivity in lymphoid malignancies.

## Methods

### Reagents

Unless stated otherwise all chemicals were purchased from Sigma-Aldrich (St. Louis, MO).

### Cell culture and treatment

CEM cell clones were derived from the original cell line CEM-CCRF, which was developed from cells of a pediatric patient with T-cell ALL. CEM-C1-15 is a Dex-resistant sub-clone obtained without selective pressure from its parental resistant clone, CEM-C1. CEM-C1-6 is a spontaneous revertant to sensitive from CEM-C1 [27]. Clones CEM-IV B9 and RPMI II E6 were chosen from a subpopulation of CEM-C1-15 or RPMI 8226 cells treated with AZA. CEM-C7-14 is a subclone of CEM-C7; both are naturally Dex-sensitive. Molt-4 and RPMI 8226 cells were from the American Type Tissue Collection (ATCC, Manassas, VA). CEM cells were grown in RPMI 1640 (Cellgro, Mediatech Inc. Herndon, VA) supplemented with 5% fetal bovine serum (FBS) (Atlanta Biologicals, Lawrenceville, GA). Both Molt-4 and RPMI 8226 cells were propagated in RPMI 1640 supplemented with 10% FBS, 10 mM Hepes buffer (Cellgro), and 1 mM sodium pyruvate. All cells were grown in a tissue culture incubator in a humidified atmosphere at 37°C, 95% air / 5% CO_2_ and subcultured regularly to ensure logarithmic growth. To evaluate Dex sensitivity, cells were treated with either ethanol vehicle (control) or concentrations of Dex ranging from 1 nM to 4 μM. For experiments examining the contribution of p38 MAPK, cells were pre-treated for 1 hour with DMSO vehicle or 1 μg/ml SB (p38 inhibitor) for CEM cells or 2 μg/ml SB for Molt-4 and RPMI 8226 cells before adding 1 μM Dex. Cells were enumerated and sized, with viability determined by Trypan blue exclusion assay, using a Vi-cell automated counter (Beckman Coulter, Fullerton, CA). For evaluation of acute demethylation, Molt-4 cells were exposed to 100 nM AZA for 24 hours prior to adding 1 μM Dex.

### Derivation of clones and analysis of Dex sensitivityciv

Preliminary dose-response experiments established the optimal concentration of AZA for production of Dex sensitivity. Consequently, CEM-C1-15, Molt-4, or RPMI 8226 cells were incubated for 48 hours in 100 nM AZA or an equal volume of its DMSO vehicle for controls, after which time the cells were pelleted by centrifugation (Beckman Coulter, Allegra 6R table-top centrifuge) at 200 × g for 5 minutes at room temperature, their supernatant medium aspirated, and resuspended in 10 mls of sterile phosphate-buffered saline (PBS) (Cellgro). Cells were pelleted as before, resuspended in fresh growth medium without AZA, and allowed to recover for 5 days. The samples were cloned by serial dilution to single cells in RPMI 1640 supplemented with 10% FBS and an insulin-transferin-selenium supplement cocktail. Cloning medium for Molt-4 and RPMI cells also contained 10 mM Hepes and 1 mM sodium pyruvate. Approximately 1 month after cloning, cell populations from each clone were grown to about 10^5^ cells per ml and evaluated for Dex sensitivity by exposure ± 1 μM Dex for 96 hours. At that time, cells were counted and evaluated for Trypan Blue dye exclusion, size and morphology (Vi-cell, Beckman). Two calculations were performed: 1.% apoptosis in the residual whole-cell population = % Trypan Blue positive cells in Dex-treated population munus % TB positive cells in Vehicle-treated Control population; and 2. “growth represson” = (100-% Trypan Blue neg cells/total cells, Dex/Control).

### Analysis of cell death

The Guidelines published in 2009 in Cell Death and Differentiation were followed: “…investigators need to combine at least two distinct methods,…and perform, whenever possible, long-term survival assays…” [43]. Accordingly, cells were evaluated in 4 ways- by phase contrast light microscopy, by vital dye exclusion and cell size- with on-screen visualization of cultures (Vi-cell, Beckman), by flow cytometry, and by multiday cell cultures following total and viable cells over time.

### Clonogenicity

RPMI 8226 cells were incubated with 100 nM AZA or DMSO vehicle for 40 hours before addition of ethanol vehicle or 1 μM Dex for an additional 96 hours. Cells were then counted and immediately serially diluted in multi-well dishes to initial average densities of 1000, 100, 10, or single cells per well. After 2 weeks they were evaluated by Trypan blue exclusion assay for wells with live cells and viable cells per well enumerated.

### Cytosine extension assay

The methylation state of cytosines within genomic DNA was analyzed using a cytosine extension method [59]. Cellular genomic DNA was extracted from logarithmically growing CEM-C1-15, CEM-IV B9, RPMI 8226, RPMI-II E6, or Molt-4 cells treated with DMSO vehicle or 100 nM AZA for 48 hours using a Qiagen DNeasy mini-prep kit (Qiagen, Valencia, CA). Residual RNA contamination was eliminated by digestion with 400 μg of RNase A. Purified DNA was quantified and 2 μg was digested overnight at 37°C using 8 units of the methylation-sensitive restriction enzyme Hpa II (New England Biolabs, Ipswich, MA). Extension was performed by combining 0.25 μg of digested DNA in the presence of ^3^H deoxycytidine triphosphate (dCTP) (53.0 Ci/mmol, Perkin Elmer, Waltham, MA), 0.25 units of TAQ polymerase (New England Biolabs), and buffer supplied with the enzyme for 1 hour at 56°C. Samples were subsequently placed on ice and duplicate 10 μl aliquots were bound onto Whatman DE-81 ion exchange filter papers (Whatman Inc., Florham Park, NJ) using a vacuum manifold. Filters were washed 3 times with 0.5 ml room temperature PBS, air dried, submerged in 3 mls of Scintiverse scintillation fluid (Fisher Scientific, Fair Lawn, NJ), vigorously vortex mixed for 15 seconds, allowed to recover for 1 hour, and evaluated using a Beckman scintillation counter (Beckman Coulter).

### Propidium iodide staining and flow cytometry

CEM-C7-14, CEM-C1-6, CEM-C1-15, CEM-IV B9, RPMI 8226, and RPMI-II E6 cells were diluted to a starting density of 5×10^4^ viable cells/ml and treated with ethanol vehicle (control) or 1 μM Dex for 96 hours. Molt-4 cells were diluted to 5×10^4^ viable cells/ml and treated with ethanol/DMSO vehicle (control), 1 μM Dex alone, or pre-treated for 24 hours with 100 nM AZA after which time 1 μM Dex or ethanol vehicle was added for an additional 72 hours. Cell nuclear suspensions were prepared, stained by PI, and analyzed by flow cytometry in the UTMB Cytometry Core Lab as described [18].

### Quantitative, real-time PCR of mRNA transcripts

TaqMan® real-time PCR assay and Taqman One-Step RT-PCR reagents (Applied Biosystems, Foster City, CA) were used to detect mRNA transcripts. The primers and Taqman probes used for hGR transcripts (1A3, 1C and 1D), c-Myb and PU.1 were described previously [52]. For measuring PGC-1a mRNA transcripts, 5′-AGAGACAAATGCACCTCCAAAAA-3′ (forward), 5′-6-FAM-AAGTCCCACACACAGTCGCAGTCACAA-TAMRA-3′ (Taqman probe) and 5′-AAAGTTGTTGGTTTGGCTTGTAAGT -3′ (reverse) were synthesized (Integrated DNA Technologies, Inc., San Diego, CA). The RT-qPCR reactions were measured on an ABI Prism 7900H Sequence Detection system (Applied Biosystems) and the results were normalized by endogenous 18S rRNA of each sample. The experiment was repeated and the results were pooled for statistical significance analysis (paired-sample t-test). Less than 5% (p < 0.05) of possible difference was considered significant.

### Immunochemical analysis

Logarithmically growing CEM-C7-14, CEM-C1-6, CEM-C1-15, and CEM-IV B9 cells were diluted to a starting density of 4×10^5^ viable cells/ml and treated for 16 hours with 100 nM Dex (GR blot), 1 μM Dex (p38 blot), or ethanol/DMSO vehicle (control). Evaluation of GR phospho-S211, total GR, phospho-p38, total p38, and actin by immunoblot was carried out as previously described [17,18].

### GR transactivation assay

Logarithmically growing CEM-C1-6, CEM-C1-15, or CEM-IV B9 cells were collected by centrifugation, washed with 10 ml of sterile 37°C PBS and recollected. The cells were resuspended to a density of 1×10^7^ viable cells/ml in serum-free 37°C RPMI 1640 containing 1.25% DMSO. Aliquots (400 μl) of the suspension were placed into 0.4 cm gap electroporation cuvettes (Bio-Rad, Hercules, CA) containing 15 μg of GRE-dependant mouse mammary tumor virus (MMTV) luciferase reporter vector phh-Luc (ATCC) prepared using a Qiagen maxi-prep kit (Qiagen). Cuvettes were electroporated using 975 μF and 270 V with a Gene Pulser II (Bio-Rad). Electroporated cells were diluted in 4 ml (per cuvette) of RPMI 1640 supplemented with 5% FBS and 1.25% DMSO and recultured. Twenty-four hours after electroporation, cellular debris was removed by filtering through a 70 μm nylon cell strainer (BD Biosciences (Falcon), Two Oak Park, Bedford, MA) and cells were pelleted, resuspended in fresh RPMI 1640 containing 5% FBS, and viable cells were quantified by hemacytometer using Trypan blue exclusion. The cultures were diluted to 2.5×10^5^ viable cells/ml in RPMI 1640 supplemented with 5% FBS, treated with ethanol vehicle (control) or 1 μM Dex, divided into 500 μl triplicate aliquots for each treatment, and placed in a 48-well tissue culture plate (Costar, Cambridge, MA). Twenty-four hours after Dex treatment, cells were pelleted and washed with PBS. Luciferase activity was evaluated by lysing cell pellets in reporter lysis buffer (Promega, Madison, WI), 1 round of freeze-thaw at -80°C to 37°C, and activation of enzymatic activity using luciferase substrate (Promega). Relative light units (RLU) were read using a Monolight luminometer (BD Biosciences). RLUs were normalized to μg of protein in the cellular lysate through use of a BCA protein assay (Pierce, Rockford, IL).

### High Throughput DNA sequencing

Total DNA from cells of C1-15, C7-14, and post-demethylation clone C1-15 IV B9 was fragmented by sonication into pieces of about 200 bp. DNA preparations were obtained representing DNA enriched for non-methylated or methylated by an immunoprecipitation procedure according to manufacturer’s protocols. . These fractions were sequenced in 36 bp fragments (reads) using an Illumina GA IIx instrument, following the manufacturer’s protocols. Quality assessment, trimming, and filtration of low quality reads were performed using our Slim-Filter software application [60]. The resulting high quality 31 bp reads were mapped to the reference human genome, build 37.3, using our in-house software. The ratios of experimental to theoretical reads at each position were computed to “normalize” the results, allowing comparisons of samples. The relative methylation status of all autosomes was determined, and the coverage across the chromosomes was calculated using a non-overlapping window size of 5000 bp. The raw sequencing data have been deposited as FASTQ files in SRA data base, identifiable under PRJNA209577. For more specific data, please contact the Corresponding Author.

### Pyrosequencing

Primers were designed using PyroMark Assay Design software version 2.0 (Qiagen). Pyrosequencing was carried out on PyroMark Q24 (Qiagen). Depending on the direction of sequencing, one of the PCR primers was biotinylated at the 5-end. All primers used for amplification of the GR proximal promoter are listed below in Table 3.

**Table 3:** Primers for amplification of GR proximal promoters

hGR-pro-1d	Forward:	5′-Biosg-ATTAAGGAAGGAAGGTTTAGGTTATT-3′
	Reverse:	5′-AAACTACCCCAACTCCACCTAATCCTA-3′
	Sequencing:	5′-CCACTCTCTCACCTC-3′
hGR-1D	Forward:	5′-AGGGAATAGTTTATTTTTGAGAATTAAGGA-3′
	Reverse:	5′-Biosg-AATAACATCCCTTACCAACTCCTAACAC-3′
	Sequencing:	5′-TTTTGAGAATTAAGGAAGGA-3′
hGR-1C	Forward:	5′-Biosg-GGATTTTGCGGGTGGAAGGAG-3′
	Reverse:	5′-ACAACCCCCCCTTTCTCCATAAATA-3′
	Sequencing:	5′-CCCTATTTAAAAAAATCTCCCATTA-3′
hGR-1C1	Forward:	5′-GGGAGTGGGGGTAGGGAT-3′
	Reverse:	5′-Biosg-CCTTCCACCCACAAAATC-3′
	Sequencing 1:	5′-AGTTGTTAAGAGTTATTAATAGG-3′
	Sequencing 2:	5′-GGGGGTGGAGTGGGA-3′
hGR-1C1C2	Forward:	5′-GGATTTTGTGGGTGGAAGG-3′
	Reverse:	5′-Biosg-AAATAAACAACCCCCCCTTTCTCCATAA-3′
	Sequencing:	5′-TTGTGGGTGGAAGGA-3′
hGR-1C2C3	Forward:	5′-AGGGGTTTTTTTTTTATTTATGGAGAA-3′
	Reverse:	5′-Biosg-CCCACTCCCCCAAACTAATAAAAATTTATA-3′
	Sequencing:	5′-TTTTTATTTATGGAGAAAGGG-3′

## Abbreviations

AZA: 5 aza-2’ deoxycytidine; AF1: Activation function 1 domain of the glucocorticoid receptor; ALL: Acute lymphoblastic leukemia; CpG islands: cytosine/guanidine repeats; dCTP: deoxycytidine triphosphate; Dex: Dexamethasone; DMSO: Dimethyl sulfoxide; DPM: Decays per minute; FBS: Fetal bovine serum; FDA: Food and drug administration; GC: Glucocorticoid; GR: Glucocorticoid receptor; GRE: Glucocorticoid-response element; MAPK: Mitogen-activated protein kinase; MMTV: Mouse mammary tumor virus; PBS: Phosphate-buffered saline; PI: Propidium iodide; PKA: Protein kinase A; S211: Serine 211 of the glucocorticoid receptor; SB: SB203580.

## Competing interests

All authors declare that they have no competing interest.

## Authors’ contributions

EBT conceived the idea of this research, directed its progress, and edited/wrote the ms. AM carried out cell biology and biochemical experiments and wrote the original draft. CG, WV performed GR promoter use and GR cofactor use experiments. JRS worked under WV’s direction. YF, GG, MS, WW carried out and interpreted the next-gen DNA sequencing. YB, LS were responsible for pyrosequencing. All authors read and approved the final manuscript.

## Authors’ information

EBT has over 50 years’ experience in the use of cell lines to study GC actions and their applications to clinical situations. AM is experienced in cell culture, molecular biology and biochemistry techniques. WV has intensively studied the use of GR promoters for many years; CG, now an independent molecular biologist, studied and worked under WV’s direction. YF is a leader in next-gen DNA sequencing; under his direction GG, MS carried out the relevant experiments. WW, an expert in application of next-gen sequencing to mitochondria, helped analyze the GR promoter data. LS is an expert in the chemistry and biology of DNA damage; JY is an expert in pyrosequencing, in the LS lab.
